# Interfacial and Rheological Characterization of High Acyl Gellan Gum–Sodium Caseinate Emulsions Under Varying pH Conditions

**DOI:** 10.3390/foods15122078

**Published:** 2026-06-08

**Authors:** Xingfen He, Yuecheng Meng, Bin Wang

**Affiliations:** 1 College of Food Science and Engineering, Gansu Agricultural University, Lanzhou 730070, China; 2 School of Food Science and Biotechnology, Zhejiang Gongshang University, Hangzhou 310018, China; mengyc@zjsu.edu.cn; 3 College of Forestry, Gansu Agricultural University, Lanzhou 730070, China; wangbin@gsau.edu.cn

**Keywords:** high acyl gellan gum, sodium caseinate, complex coacervation, pH-responsive emulsion, rheological properties

## Abstract

Sodium caseinate (SC)-stabilized emulsions are highly susceptible to flocculation and phase separation near the protein isoelectric point (pI), limiting their application in acidified food systems. In this study, high acyl gellan gum (HA) was introduced to construct pH-responsive protein–polysaccharide complexes to modulate the interfacial assembly and stability of SC emulsions. Results demonstrated that HA interacts with SC primarily through electrostatic attraction and multi-site hydrogen bonding. This interaction induces protein conformational rearrangement and, as evidenced by combined structural and computational analyses, facilitates the assembly of a denser, interconnected composite network. The formation of HA–SC complexes significantly enhanced interfacial adsorption, reduced oil–water interfacial tension. Rheological and microrheological analyses revealed the composite system formed an elasticity-dominated weak gel network, restricting droplet mobility and suppressing aggregation. Consequently, HA–SC emulsions exhibited markedly improved pH tolerance and physical stability compared to SC-only emulsions, particularly near the pI, evidenced by reduced droplet size, lower Turbiscan stability indices, and more homogeneous microstructures. Crucially, utilizing a well-defined mechanistic model of fixed HA and SC concentrations, this study quantitatively links molecular interactions, interfacial network reconstruction, and macroscopic emulsion stability across a broad pH continuum. Rank-correlation analysis of pH-resolved descriptors shows the molecular charge state co-varies monotonically with the interfacial network and macroscopic stability, and is inversely coupled to droplet mobility. These findings provide new insights into protein–polysaccharide interfacial engineering, establishing the essential physical-stability foundation for the future rational design of acid-tolerant food emulsions and functional delivery systems.

## 1. Introduction

Oil-in-water (O/W) emulsions are central to a large fraction of liquid and semi-solid food products, and they remain one of the most accessible platforms for delivering lipophilic bioactives such as fat-soluble vitamins, carotenoids, polyphenols and ω-3 fatty acids into aqueous matrices [1,2]. Their performance, however, depends entirely on the interfacial layer that surrounds each oil droplet, and this thin layer is unusually sensitive to environmental conditions such as pH, ionic strength and temperature. Of these, pH is the most demanding variable for protein-stabilised systems. Real food matrices and the gastrointestinal tract together cover a pH window of roughly 2 to 9, and most proteins approach or pass through their isoelectric point (pI) somewhere along the way [3].

Sodium caseinate (SC) is among the most widely used food protein emulsifiers, particularly in dairy beverages, cream liqueurs, coffee whiteners and infant formulations [4]. Its four constituent caseins (α_s1_-, α_s2_-, β-, κ-) form a flexible, partially disordered assembly that adsorbs rapidly to the oil–water interface and provides combined electrostatic and steric stabilisation against droplet coalescence. It is well documented that at pH values close to the SC pI (~4.6), the net charge on adsorbed caseinate falls toward zero, electrostatic repulsion between droplets is lost, and extensive flocculation, creaming and phase separation set in [5]. This single weakness still constrains the use of SC in acidic functional beverages, fermented dairy analogues and many plant-based formulations.

The most widely used strategy for extending the operational pH window of SC-based emulsions is pairing the protein with an anionic polysaccharide to form pH-driven electrostatic complexes. This association stabilizes droplets either by providing an additional electrosteric barrier or by building a viscoelastic continuous phase that kinetically traps the dispersed oil [6,7,8]. High acyl gellan gum (HA) has emerged as a particularly attractive structural partner for SC in this context. As a linear, anionic exopolysaccharide featuring a dense array of carboxyl, hydroxyl, and acyl (L-glyceryl and acetyl) groups, HA maintains a uniform negative charge density across the entire food-relevant pH range while providing multiple hydrogen-bond donors and acceptors [9,10]. These structural features ensure strong electrostatic and hydrogen-bonding complementarity with SC once the protein acquires a net positive charge below pH 4.6. Furthermore, HA forms soft, thermo-reversible weak gels at low concentrations and is highly acid-tolerant, making it an ideal stabilizer for acidic formulations without compromising mouthfeel or clarity [10,11].

A growing body of work highlights the practical value of HA in stabilizing casein-based emulsions. Previous studies demonstrate that HA enhances macroscopic stability primarily through two mechanisms: modifying bulk-phase rheology by increasing continuous-phase viscosity and forming a viscoelastic matrix [12,13], and reinforcing the oil–water interface via electrostatic complexation with SC at acidic pH [14]. Additionally, HA has shown potential in improving gastric stability and interfacial properties in various lipid and protein systems [15,16]. Despite this momentum, a comprehensive multi-scale understanding remains elusive. First, the continuous pH-induced phase transitions of HA-SC systems, which span from soluble and insoluble complexation to coacervation and redispersion, have not been systematically linked to their interfacial and bulk rheological properties. Second, the molecular interactions driving HA–SC assembly are largely inferred from ensemble spectroscopy, lacking direct insight into single-chain bridging behaviors and secondary structure rearrangements. Consequently, a coherent mechanistic picture connecting chain-level molecular events, complex structural reorganization, and macroscopic emulsion stability has yet to be fully established.

While recent progress has been made in understanding HA–SC systems, previous studies have largely focused on localized interfacial phenomena or specific environmental stresses. For instance, Farooq et al. elegantly demonstrated the interfacial adsorption kinetics of SC/HA complexes, which does not cover the complete pH spectrum. Similarly, our previous work evaluated the macroscopic rheological properties of these emulsions strictly under salt ion stress [17]. Despite this momentum, a comprehensive mechanistical link spanning from atomistic molecular interactions to microscopic droplet dynamics and macroscopic stability across a full food-grade pH continuum (3.0–11.0) has remained unresolved. To address these gaps, we followed HA–SC complexation across the full pH range of practical interest (3.0–11.0) and connected the molecular, interfacial and macroscopic length scales within one study. All-atom molecular dynamics simulations were used to capture HA–SC bridging and the associated hydrogen-bond network, and the predictions were tested against FTIR, XRD, DSC and TGA measurements of secondary structure and thermal behaviour. Pendant-drop interfacial tension was used to follow biopolymer adsorption at the oil–water interface, and small-amplitude oscillatory shear rheology was employed to investigate the viscoelasticity of the continuous phase, while diffusing-wave- spectroscopy (DWS) microrheology was utilized to extract droplet-level dynamics, such as mean-square displacements and microviscosity, which remain unresolved by bulk rheometry [18]. Macroscopic stability was then characterised by Turbiscan multiple-light-scattering analysis, particle-size distribution, confocal laser scanning microscopy and visual storage observation. Rather than screening a broad compositional space to identify an optimum formulation, our intent was to utilize a strictly defined model system (fixed at 0.2% *w*/*v* SC and 0.125% *w*/*v* HA) to build a comprehensive overview. By holding the compositional space narrow, we explored how molecular events, network formation, and emulsion stability are linked across a broad pH continuum to assess how far HA can extend the operational pH window of SC-stabilised emulsions. Establishing this physical and colloidal stability across a broad pH continuum serves as a necessary prerequisite for any downstream functional applications; subsequently, evaluating their in vitro gastrointestinal stability and bioaccessibility remains a key next step to validate their potential as delivery systems.

## 2. Materials and Methods

### 2.1. Materials

High acyl gellan gum (HA, LT100-P) was supplied by CP Kelco Inc. (Shanghai, China). Sodium caseinate (SC), Oil Red O, Nile Red and fluorescein isothiocyanate (FITC) were obtained from Sigma-Aldrich (St. Louis, MO, USA). Analytical-grade 1,2-propanediol and hydrochloric acid were purchased from Macklin Biochemical Co., Ltd. (Shanghai, China), and the ProClean 150 antimicrobial agent was from Beyotime Biotechnology Co., Ltd. (Shanghai, China). Refined sunflower oil was bought from a local supermarket in Zhejiang, China. All other reagents were of analytical grade. Deionised water (≥18.2 MΩ·cm) prepared with a Milli-Q system (Merck Millipore, Darmstadt, Germany) was used throughout.

### 2.2. Turbidity Measurement

The turbidity of HA–SC dispersions was monitored at 600 nm and 25 °C on a UV-2000 spectrophotometer (Unico, Shanghai, China) using 1 cm path-length quartz cuvettes, with deionized water adjusted to the corresponding pH using 1 M HCl or NaOH used as the blank. To prevent electrostatic screening effects, no standard buffer systems or background salts were utilized, ensuring the overall ionic strength remained minimal across all pH conditions [19]. Turbidity (τ, cm^−1^) was calculated as:

(1) $$\left(T\right)=-\frac{1}{L}\ln\left(\frac{I}{I_{0}}\right)$$

where *L* is the path length, *I* and *I*_0_ represent the transmitted and incident light intensities, respectively.

### 2.3. ζ-Potential and Particle Size Determination

The ζ-potential of HA–SC mixtures was determined on a Zetasizer Nano ZS90 (Malvern Instruments, Worcestershire, UK) following the protocol of Duan et al. [20]. Samples were diluted to 1 mg mL^−1^ using deionized water adjusted to the matching pH with 1 M HCl or NaOH, and a 650 μL aliquot was loaded into a folded capillary cell (DTS1070). Each measurement was carried out at 25 °C after a 120 s equilibration. The hydrodynamic size of the complexes was acquired on the same instrument under identical conditions, with a particle refractive index of 1.461, an absorption index of 0.01 and a dispersant refractive index of 1.330 (water). Prior to measuring emulsions, samples were diluted in pH 7.0 adjusted deionized water to suppress multiple scattering.

The droplet size distribution of the emulsions was further determined by laser diffraction on a Mastersizer 3000 (Malvern Instruments, Malvern, UK) with water as the dispersant [17]. The particle and dispersant refractive indices were 1.461 and 1.330, and the particle absorption index was 0.000. The volume-weighted (*D*_43_) and surface-weighted (*D*_32_) mean diameters were calculated as:

(2) $$D_{32}=\frac{\sum n_{i}d_{i}^{3}}{\sum n_{i}d_{i}^{2}}$$

(3) $$D_{43}=\frac{\sum n_{i}d_{i}^{4}}{\sum n_{i}d_{i}^{3}}$$

where *n_i_* is the number of droplets of diameter *d_i_*.

### 2.4. Molecular Dynamics Simulation

#### 2.4.1. Model Construction

Because no full-length crystal structure of bovine casein is currently deposited in the Protein Data Bank, three-dimensional models of the α_s1_- (UniProt P02662), α_s2_- (P02663) and β-casein (P02666) subunits were generated using AlphaFold2 (https://github.com/google-deepmind/alphafold (accessed on 3 June 2026)) [21]. As caseins are intrinsically disordered proteins, AlphaFold2 returns low per-residue confidence (pLDDT < 50) over extended portions of each chain; the resulting models were therefore treated as conformational starting points rather than as well-folded structures. Prior to assembly, the 15-residue N-terminal secretion signal peptide was removed from each subunit, as it is cleaved in vivo and is not present in mature casein. In addition, the extreme N- and C-terminal segments of very low confidence (pLDDT < 50) were truncated to prevent non-physical excursions of these unconstrained tails from artifactually entangling with the polysaccharide and unnecessarily enlarging the solvation box: residues [1–14] and [190–199] of αs1-casein, [1–15] and [190–207] of αs2-casein, and [1–20] and [200–209] of β-casein (mature-chain numbering). The trimmed subunits were then assembled into a simplified ternary aggregate, which was used as a qualitative model of localized non-covalent HA–casein interactions rather than as a quantitative descriptor of the proteins’ full conformational behavior [22]. A HA fragment with a degree of polymerisation of 16 was built in ChemOffice Professional 16 (PerkinElmer, Waltham, MA, USA), and its CHARMM-compatible topology file was generated through the CHARMM-GUI server (https://www.charmm-gui.org (accessed on 3 June 2026)) [23].

#### 2.4.2. Molecular Dynamics Simulation Procedure

All-atom MD simulations were performed in GROMACS 2020.7 using the CHARMM36 force field [24,25]. The composition of each simulation system is summarised in Table 1. Hydrogen atoms were first added to the protein, the HA–SC complex was placed at the centre of a cubic box, and the box was solvated with TIP3P water with a minimum solute–box distance of 2.0 nm. Sodium counterions were added to neutralise the system. Energy minimisation was carried out for up to 50,000 steps using the steepest-descent algorithm to remove unfavourable contacts. The system was then equilibrated at 298.15 K in the NVT ensemble (50,000 steps, 2 fs time step, position restraints on heavy atoms), followed by NPT equilibration at 298.15 K and 1 bar (50,000 steps, 2 fs). Production runs of 100 ns were finally performed in the NPT ensemble under the same conditions, with frames written every 100 ps. Periodic boundary conditions were applied in all three directions. While this 100 ns duration is concise for observing bulk macroscopic assembly, it is well-established as adequate for capturing the localized molecular docking, conformational rearrangement, and hydrogen-bond network formation between specific biopolymer fragments at the single-chain level. Bonds involving hydrogen were constrained with the LINCS algorithm [26]. Temperature was controlled with a V-rescale thermostat (τ_T_ = 0.1 ps), pressure with a Parrinello–Rahman barostat (τ_P_ = 1.0 ps), and long-range electrostatics were treated with the particle-mesh Ewald (PME) method. From the production trajectories, the root-mean-square deviation (RMSD) of the protein backbone was used to monitor structural stability, and the number of intermolecular hydrogen bonds between casein and HA was extracted to characterise their non-covalent association. It is important to acknowledge the inherent assumptions and limitations of the constructed MD model. The simulated system is a highly simplified representation of the experimental microenvironment. Specifically, the model employs truncated casein subunits assembled into an interior ternary aggregate lacking κ-casein, utilizes a single HA fragment with a limited degree of polymerization (DP = 16), and operates under a fixed neutralizing ionic state (Na^+^) without explicit parameterization for the dynamic pH variations (pH 3.0–11.0) applied in our macroscopic experiments. Furthermore, the simulation was conducted in a purely aqueous phase, which does not fully capture the amphiphilic rearrangement of caseins at the oil-water interface. Consequently, these simulations are not designed to serve as quantitative predictors of the complex, pH-dependent phase behaviors or macroscopic emulsion stability. Rather, the MD model is intended strictly as a qualitative illustration to explore possible localized non-covalent docking, spatial bridging, and multi-site hydrogen-bonding motifs at the single-chain level.

### 2.5. Fourier Transform Infrared (FTIR) Spectroscopy

Lyophilised HA–SC complexes were ground in an agate mortar, and approximately 2 mg of powder was blended with 198 mg of dry KBr and pressed into a transparent pellet. Spectra were recorded on a Fourier-transform infrared spectrometer (iNicolet iS50; Thermo Fisher Scientific, Waltham, MA, USA) at 25 °C from 4000 to 500 cm^−1^ at a resolution of 4 cm^−1^, with 32 scans co-added per spectrum [27]. Curve fitting and deconvolution of the amide I band were performed in PeakFit V4.12 (Systat Software, San Jose, CA, USA).

### 2.6. X-Ray Diffraction (XRD) Analysis

XRD patterns of the lyophilised samples were collected on an X-ray diffractometer (D8 Advance; Bruker AXS, Karlsruhe, Germany) with Cu-Kα radiation (λ = 0.154 nm), operated at 40 kV and 30 mA. Samples were scanned over a 2θ range of 5–40° with a step size of 0.02° and a scan rate of 1° min^−1^ [28].

### 2.7. Thermal Stability

#### 2.7.1. Differential Scanning Calorimetry (DSC)

About 3 mg of lyophilised sample was sealed in an aluminium pan and scanned on a differential scanning calorimeter (DSC 250; TA Instruments, New Castle, DE, USA) from 20 to 200 °C at 10 °C min^−1^ under a nitrogen flow of 50 mL min^−1^. The peak denaturation temperature was extracted from the resulting thermogram using the instrument software (TriOS software v5.1) [29].

#### 2.7.2. Thermogravimetric Analysis (TGA)

Approximately 10 mg of lyophilised sample was loaded into a sample pan and heated on a thermogravimetric analyzer (TGA 550; TA Instruments, New Castle, DE, USA) from 50 to 500 °C at 20 °C min^−1^ under a 50 mL min^−1^ N_2_ purge [30].

### 2.8. Dynamic Interfacial Tension

The dynamic oil–water interfacial tension of SC and HA–SC dispersions against sunflower oil was measured at 25 °C on an optical contact-angle goniometer (OCA 25; DataPhysics Instruments GmbH, Filderstadt, Germany) fitted with an oscillating-drop module (ODG-20). An aqueous droplet was extruded from a J-shaped polymer needle into the surrounding oil phase, and the interfacial tension (γ) was extracted from the drop contour by fitting the Young–Laplace equation [31]. Each curve is the average of three independent drops.

### 2.9. Emulsion Preparation and pH Stability Evaluation

The oil phase was prepared by dissolving Oil Red O (0.5%, *w*/*v*) in sunflower oil under magnetic stirring in the dark until full dissolution; the dye served as a tracer for visualising the lipid phase. The optimal HA concentration (0.125%, *w*/*v*) was selected based on a preliminary concentration screening study conducted prior to this work, which demonstrated that 0.125% HA exhibited the most pronounced improvement in emulsion stability, as detailed in our previous publication [17]. A fixed SC concentration of 0.2% (*w*/*v*) was employed throughout this study, as this concentration is well-established in the literature to achieve complete interfacial coverage and stable emulsion formation while still allowing sufficient sensitivity to detect HA-mediated stabilization effects. The aqueous phase (pH 7.0) was prepared as a HA-SC mixed solution containing a fixed concentration of SC (0.2%, *w*/*v*) and HA (0.125%, *w*/*v*). To ensure complete hydration and homogeneous dispersion, the aqueous phase was allowed to equilibrate under continuous magnetic stirring at 25 °C for 2 h prior to emulsification. Aqueous and oil phases were combined at 9:1 (*v*/*v*), giving a final oil fraction of 10% (*v*/*v*), and pre-emulsified with a high-shear homogenizer (Ultra-Turrax T25; IKA-Werke GmbH, Staufen, Germany) at 10,000 rpm for 2 min. The pre-emulsion was then passed three times through a high-pressure homogenizer (PandaPlus 2000; GEA Niro Soavi, Parma, Italy) at 200 bar to obtain the fine emulsion, and ProClean 150 (0.1%, *v*/*v*) was added as an antimicrobial agent [32]. Freshly prepared emulsions were gradually adjusted dropwise to target pH values (3.0, 5.0, 7.0, 9.0, and 11.0) using 1 M HCl or NaOH solutions under gentle magnetic stirring to avoid localized pH shock. The pH-adjusted emulsions were then transferred into sealed transparent glass vials and allowed to re-equilibrate undisturbed at 25.0 ± 0.5 °C for 24 h prior to subsequent microstructural, rheological, and stability analyses (with the exception of initial 0 h visual storage observations). High-concentration acid and base were deliberately selected to minimize the titration volume, thereby introducing only negligible amounts of Na^+^ or Cl^−^ counterions. Consequently, while the ionic strength was not strictly maintained at a constant level across all samples with background salts, the overall ionic strength remained extremely low. This ensured that the observed multiscale interfacial assembly and emulsion phase behaviors were predominantly driven by pH-induced macromolecular charge transitions rather than electrostatic screening effects. The pH-adjusted emulsions were transferred into sealed transparent glass vials, and digital images were captured at 0 h and 24 h with a digital camera (EOS 80D; Canon Inc., Tokyo, Japan) to observe the storage stability of the emulsions.

### 2.10. Confocal Laser Scanning Microscopy (CLSM)

The microstructure of the emulsions was imaged on a confocal laser scanning microscope (TCS SP8; Leica Microsystems, Wetzlar, Germany) using a 100× oil-immersion objective. Stock solutions of Nile Red (0.02%, *w*/*v*) and FITC (0.10%, *w*/*v*) were prepared in 1,2-propanediol/water (50:1, *v*/*v*) and combined at 1:1 (*v*/*v*) immediately before use. A 40 μL aliquot of the staining mixture was added to 1 mL of emulsion and vortexed; after 15 min in the dark, a 10 μL drop was placed on a glass slide and covered with a coverslip. Images of 1024 × 1024 pixels were acquired with sequential excitation at 488 nm (FITC, protein phase; emission 500–540 nm) and 561 nm (Nile Red, oil phase; emission 580–650 nm) [20].

### 2.11. Bulk Rheology

Bulk rheological behaviour of the emulsions was characterised at 25 °C on a stress-controlled rheometer (DHR-3; TA Instruments, New Castle, DE, USA) fitted with a parallel-plate geometry (40 mm diameter, 1 mm gap). Steady-shear flow curves were recorded over a shear-rate range of 0.1–10 s^−1^ (10 points per decade). Frequency sweeps were carried out from 0.1 to 10 rad s^−1^ (10 points per decade, logarithmic) at a fixed strain of 1%, after an amplitude sweep had confirmed that this strain lay within the linear viscoelastic region [33].

### 2.12. Microrheology

Microrheological behaviour was probed by diffusing wave spectroscopy (DWS) on a DWS Research Lab instrument (LS Instruments, Fribourg, Switzerland) operating in transmission mode (red diode laser, λ = 685 nm, 45 mW). To evaluate droplet dynamics, the intensity autocorrelation function (g_2_ (τ) − 1, dimensionless), which captures the temporal fluctuations of scattered light caused by the thermal Brownian motion of the droplets, was recorded. Both the apparent particle size and the viscoelastic descriptors were extracted from this autocorrelation function. Specifically, these descriptors include the mean square displacement (MSD, Δr^2^(τ), expressed in nm^2^), which defines the average spatial extent of droplet movement over a given lag time τ. By applying the generalized Stokes-Einstein equation, the frequency-dependent microviscosity (Pa·s) was calculated to assess the local flow resistance. Additionally, the elastic and viscous indices (dimensionless) were derived to quantify the solid-like elasticity and liquid-like dissipation of the continuous composite network, respectively. Acquisition and echo time were both set to 30 s, and measurements were performed at 25 °C. Samples were loaded into 2 mm path-length glass cuvettes and equilibrated for 60 s before each acquisition. Data were processed with the manufacturer’s software (DWS ResearchLab Software, version 3.4) [34].

### 2.13. Multiple Light Scattering

The kinetic stability of the emulsions was monitored on a Turbiscan Lab (Formulaction, Toulouse, France), which records backscattered and transmitted light along the height of the sample tube using the principle of multiple light scattering [35]. Samples were equilibrated undisturbed for 1 h prior to measurement and then scanned at 30 min intervals for 12 h at 25 ± 0.5 °C. The Turbiscan Stability Index (TSI) was calculated using the manufacturer’s software (Turbisoft software, version 2.2).

### 2.14. Statistical Analysis

All measurements were performed in triplicate, and data are reported as mean ± standard error. Significant differences between means were assessed by one-way ANOVA followed by Duncan’s multiple-range test (*p* < 0.05) using SPSS Statistics 26.0 (IBM Corp., Armonk, NY, USA). To properly address the experimental factors, separate one-way ANOVAs were conducted: the effect of pH was analyzed independently within the pure SC and HA-SC groups, while the effect of HA addition was evaluated by comparing the two groups at each specific pH level. Plots were generated in OriginPro 2024 (OriginLab, Northampton, MA, USA).

## 3. Results and Discussion

### 3.1. pH-Dependent Electrostatic Assembly Behavior of HA-SC Complexes

**Figure 1:**
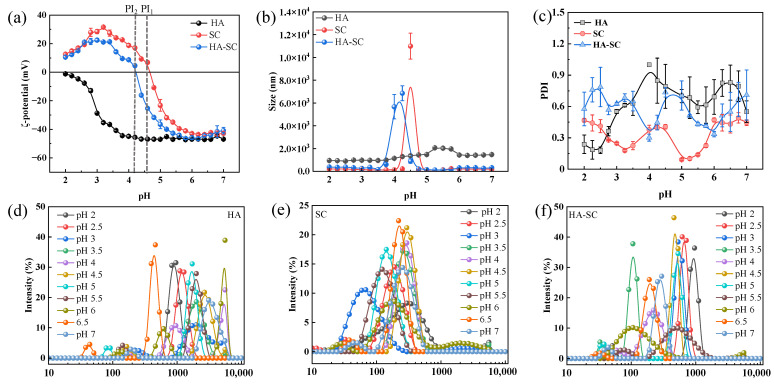
Effect of pH on the (**a**) ζ-potential, (**b**) mean hydrodynamic particle diameter, (**c**) polydispersity index (PDI), and (**d**–**f**) particle size distributions of HA, SC, and HA-SC complexes. Bars represent the standard error.

The electrostatic association between proteins and anionic polysaccharides depends strongly on solution pH because changes in the protonation state of charged groups regulate intermolecular attraction and colloidal stability [36]. As shown in Figure 1a, the ζ-potential of sodium caseinate (SC), High acyl gellan gum (HA) and HA-SC complexes increased progressively as the pH was lowered, reflecting the gradual neutralization of carboxylate groups under acidic conditions. Native SC underwent charge reversal at around pH 4.6, in agreement with its reported isoelectric point [37]. The apparent pI of the HA-SC complex, however, shifted to a lower value of approximately 4.2, suggesting that the incorporation of HA modified the surface charge distribution of SC and partially compensated for the loss of negative charge near the protein pI [13].

The reduced shift in ζ-potential observed for the HA-SC system around the protein pI implies that HA limits extensive aggregation through a combination of electrostatic and steric stabilization. Comparable shifts in apparent pI together with improved colloidal behavior have been documented for whey protein-hyaluronic acid mixtures, in which the polyanionic polysaccharide redistributed surface charges and limited protein self-association [38].

Particle size measurements further confirmed the pH-responsive assembly of the complexes (Figure 1b,c). Between pH 6.0 and 7.0, strong electrostatic repulsion preserved relatively small particles (123–327 nm), consistent with the formation of soluble complexes. As the pH approached 5.4, the average diameter remained close to 127 nm, indicating that compact and homogeneous associates dominated the system. Below pH 5.0 the particle size increased sharply to 902 nm, marking the appearance of insoluble coacervates. The largest aggregates (~6858 nm) formed near pH 4.2, where the residual electrostatic repulsion was minimal, and the broadening of PDI confirmed the strong heterogeneity of these structures.

The full size distribution profiles (Figure 1d–f) provided additional insight into this dynamic process. At pH 5.5, a bimodal distribution appeared, consistent with the coexistence of free biopolymers and primary intra-polymeric complexes given that both HA and SC still carried appreciable negative charges. Acidification to pH 4.0 collapsed the distribution into a broad unimodal peak, evidence of widespread inter-polymeric aggregation driven by stronger electrostatic attraction. Below pH 3.5 the distribution narrowed once more, suggesting that excessive protonation of HA carboxyl groups partially dissociated or rearranged the large coacervates. Such successive transitions among co-soluble species, soluble complexes and insoluble coacervates have also been described for whey protein-polysaccharide systems [36,38].

**Figure 2:**
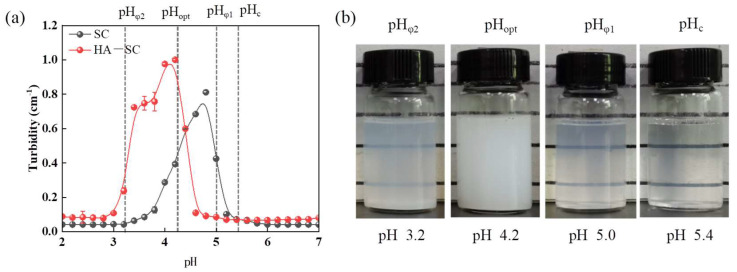
pH-dependent phase behavior of the HA–SC mixture at 25 °C: (**a**) turbidity expressed as 100 − T% as a function of pH; and (**b**) the corresponding physical phase map combining the turbidity data with photographs of the mixtures. Bars represent the standard error.

To systematically illustrate the aforementioned continuous phase transitions, a comprehensive physical phase map was constructed based on turbidity profiles and visual appearance (Figure 2). This phase map visually suggests that the incorporation of HA fundamentally altered the distribution of characteristic phase-transition pH values. For the pure SC system, phase separation occurred abruptly, rapidly reaching maximum aggregation and yielding macroscopic precipitation near its isoelectric point (pH ≈ 4.6). In contrast, the phase-transition boundaries of the HA-SC composite system exhibited a pronounced shift toward lower pH values. Specifically, between pH 6.0 and 7.0, the system predominantly existed as “soluble complexes”; as the pH dropped below 5.0, the critical phase separation point was crossed, marking the emergence of “insoluble coacervates”. Crucially, the electrical equivalence point (optimal coacervation pH), which corresponds to the strongest electrostatic attraction and maximum aggregate size, shifted significantly from pH 4.6 for pure SC to approximately pH 4.2 for the HA-SC complex. Upon further acidification below pH 3.5, excessive protonation weakened the associative interactions, driving the system back into a region of partial dissociation and “redispersion”. The modulation of these characteristic pH values in the phase map not only systematically delineates the evolutionary trajectory from co-solubility and complexation to coacervation and redispersion, but also provides compelling evidence that HA can effectively delay and regulate protein aggregation under acidic conditions.

Taken together, these observations show that pH effectively tunes the electrostatic assembly of HA and SC, driving the system from dispersed soluble complexes through dense coacervates and back again. This pH-responsive behavior establishes the structural foundation for the interfacial and rheological properties of the resulting emulsions discussed in the following sections.

### 3.2. Molecular Interactions and Structural Rearrangement in HA-SC Complexes

**Figure 3:**
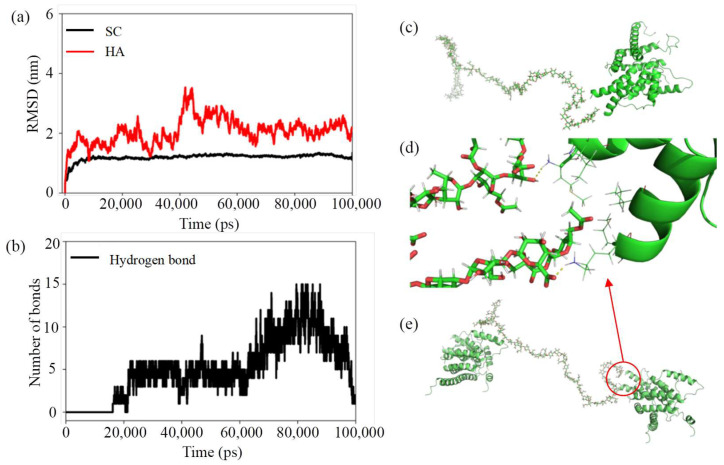
All-atom molecular dynamics (MD) analysis of the HA–SC molecular system: (**a**) time evolution of the protein-backbone root-mean-square deviation (RMSD); (**b**) number of intermolecular hydrogen bonds formed between HA and SC along the trajectory; (**c**) schematic illustration of the overall HA–SC interaction; (**d**) close-up view of the hydrogen bonds at the HA–SC contact interface; and (**e**) representative configuration in which a single HA chain bridges two SC molecules through multivalent hydrogen bonding.

To clarify the interactions responsible for HA-SC complexation, molecular dynamics (MD) simulations were combined with spectroscopic and diffraction analyses. The RMSD trajectories converged within a few nanoseconds and showed only minor fluctuations afterwards (Figure 3a). This rapid stabilization indicates that a 100 ns simulation time was sufficient for the system to reach local thermodynamic equilibrium. Therefore, rather than reflecting large-scale phase coacervation, the simulation successfully captured the localized structural stability and steady-state docking behavior of the biopolymers over the course of the observation.

The MD trajectories also revealed the formation of multiple hydrogen bonds between HA hydroxyl groups and amino acid residues on SC (Figure 3b). Owing to its extended linear conformation, MD simulations suggest the possibility of multivalent bridging, in which a single HA chain could interact with several SC molecules through hydrogen bonding (Figure 3c–e), suggesting the theoretical potential for forming a spatially interconnected network. This simulated bridging effect provides a plausible molecular basis for the restricted protein mobility and limit further self-aggregation observed macroscopically. Similar bridging behavior has been reported for peach gum–sodium caseinate systems characterized through molecular docking and structural analyses [39]. Given the structural simplifications of the simulation, most notably the absence of κ-casein, the use of a single, short HA chain, and the application of a fixed ionic environment without explicit pH titration, these computational trajectories must be interpreted strictly as qualitative evidence. They do not quantitatively represent the dynamic dissociation and complex coacervation of caseinate across the full pH continuum observed experimentally. Instead, they effectively visualize the potential microscopic mechanisms by illustrating how spatial bridging and multivalent hydrogen-bonding motifs can occur between HA chains and casein subunits. This theoretical illustration perfectly complements the localized structural rearrangements detected by our subsequent spectroscopic analyses, without overextending computational claims to the bulk phase macroscopic behavior. While the current simplified model precludes precise residue-specific quantitative mapping, these qualitative trajectories perfectly complement the localized structural rearrangements detected by our robust spectroscopic analyses (FTIR and XRD).

**Figure 4:**
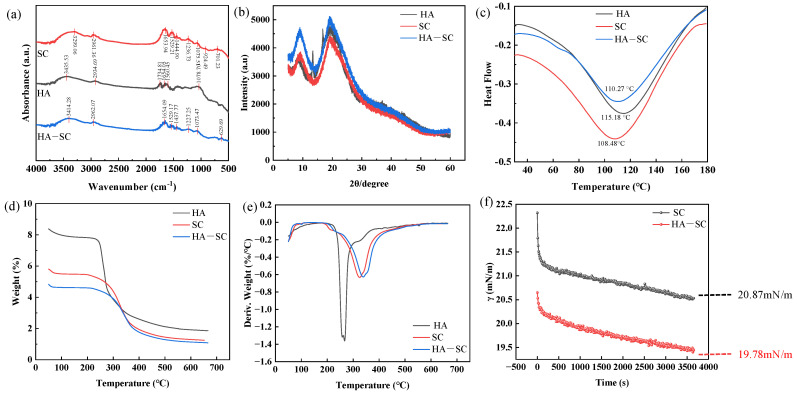
Structural and thermal characterization of freeze-dried HA, SC, and HA–SC complex coacervates: (**a**) Fourier-transform infrared (FTIR) spectra (4000–500 cm^−1^); (**b**) X-ray diffraction (XRD) patterns (2θ = 5–40°); (**c**) differential scanning calorimetry (DSC) thermograms; (**d**) thermogravimetric analysis (TGA) curves; and (**e**) the corresponding derivative thermogravimetric (DTG) curves. (**f**) Dynamic oil–water interfacial tension of the SC and HA–SC dispersions against sunflower oil at 25 °C.

The marked sensitivity of casein-based colloidal systems to subtle environmental modifications, and the analytical leverage gained by pairing dispersion-scale measurements with atomistic-level simulations, has been further substantiated by recent work on milk colloids. Blinov et al. combined photon-correlation and electroacoustic spectroscopy with quantum-chemical modelling of negatively charged C-terminal fragments of κ-casein (including Ala-Ser(P)-Pro, Ile-Glu-Ser and sialic-acid moieties) to demonstrate that different zinc-containing ligands generate markedly distinct perturbations in the hydrodynamic radius and ζ-potential of casein micelles, with the smallest changes corresponding to the chelated configuration (Zn-lysinatoriboflavinate) that minimised free Zn^2+^ release and best preserved the negative surface charge of the micelles [40]. The convergence between such ion-induced surface-charge perturbations and the pH-induced charge transitions observed in the present HA-SC system reinforces a broader principle: the casein interfacial layer behaves as a finely tuned electrostatic surface whose colloidal stability is governed by the competition between attractive contributions (charge neutralisation, ion bridging, multi-site hydrogen bonding) and repulsive contributions (electrostatic and steric). Critically, resolving these contributions in a mechanistically meaningful way requires complementary information from multiple length scales, which is why the joint deployment of dispersion- and interfacial-scale experimental probes with atomistic or quantum-chemical simulations is increasingly recognised as the preferred paradigm for elucidating protein–colloid interactions. The present work adopts this integrated strategy, coupling all-atom MD with FTIR, XRD, DSC, interfacial-tension, bulk and DWS microrheology measurements to link single-chain bridging events to macroscopic emulsion behaviour across a broad pH continuum, and the parallels with the Zn–casein system underline that this multiscale approach is broadly transferable across casein-based colloidal platforms challenged by chemically distinct environmental modifiers. FTIR analysis supported the interactions identified by MD (Figure 4a). Native SC exhibited the characteristic amide I and amide II bands at 1653.94 and 1529.21 cm^−1^, whereas HA showed a broad O-H stretching band at 3435.53 cm^−1^ together with carboxyl-related absorption [12]. Following complexation, both the hydroxyl- and amino-associated regions shifted noticeably. The disappearance of the HA acetyl peak, together with shifts in the amino and carboxyl stretching modes, indicates a distinct change in the local chemical environment and a closer conformational packing between the two macromolecules. When interpreted alongside the ζ-potential data and the multivalent bridging observed in the molecular dynamics (MD) simulations, these spectral shifts are highly consistent with the formation of an interconnected HA–SC network driven primarily by electrostatic attraction and hydrogen bonding.

Deconvolution of the amide I region also revealed a substantial conformational rearrangement of SC after complexation (Table 2). The β-sheet content of the HA-SC complex rose from 32.02% to 42.75%, while the α-helix and random-coil contents fell appreciably. While an increase in β-sheet structures is frequently an indicator of protein aggregation, in this context, it reflects the localized, controlled intermolecular association between SC and HA during complexation. Rather than simply adopting a stabilized intramolecular conformation, the multi-site hydrogen bonding encourages SC molecules to rearrange into a denser, highly interconnected structural network. This transition into a more denser packing state is characteristic of complex coacervation, representing a physically stable, aggregated composite structure [7,41].

XRD patterns supplied complementary information about the changes in the spatial packing of the biopolymers (Figure 4b). Pure SC displayed broad diffraction peaks at 2θ = 9.49° and 19.29°, while HA showed primary peaks at 2θ = 8.56° and 19.47°. The diffuse character of these reflections confirms that both biopolymers exist predominantly in an amorphous state. After complexation, the HA-SC peaks shifted to 2θ = 9.02° and 19.38° and intensified markedly (4731 and 5102 a.u., respectively), values clearly higher than those of either component alone. Rather than indicating a crystalline transition, this intensification points to a closer local association and denser packing of the macromolecular chains, consistent with the formation of a highly compact amorphous composite network [7,41]. Comparable peak enhancement has been reported for protein-polysaccharide systems in which intermolecular interactions facilitated assembly into denser, more homogeneous microstructures [42].

The structural reinforcement driven by HA-SC complexation was further corroborated by the thermal degradation and denaturation profiles of the complexes. DSC measurements indicated that the endothermic peak—corresponding to the peak denaturation temperature of the HA-SC complex—shifted to a noticeably higher value compared to that of native SC (Figure 4c), pointing to improved thermal resistance. Rather than a simple additive effect, this enhanced thermal stability stems directly from the extensive non-covalent interactions bridging the two biopolymers. The strong electrostatic attraction and multivalent hydrogen bonding, as visualized in the MD simulations, effectively cross-link the HA and SC chains, restricting the conformational mobility of the casein backbone. Furthermore, the transition toward a more compact, stabilized conformation with increased β-sheet content raises the thermodynamic energy barrier required to unfold the protein. Consequently, significantly more thermal energy is necessary to disrupt this stabilized composite network. Complementary insights were provided by the TGA and derivative thermogravimetry (DTG) curves (Figure 4d,e). While the incorporation of HA hardly altered the ultimate high-temperature degradation profile of SC, the structural stability of SC was notably improved at the low and intermediate temperatures during protein denaturation. The initial mass loss phase, typically associated with the evaporation of free and bound water, was delayed in the HA-SC complex. This suggests that the dense array of hydroxyl and carboxyl groups on the HA chains tightly binds interfacial water molecules, beneficially altering the hydration shell of the protein. During the intermediate degradation phase, which corresponds to protein depolymerization and the cleavage of side chains, the interconnected HA-SC network acted as a physical scaffold that restricted these thermal decomposition pathways. Together, these thermal analyses move beyond a macroscopic observation of stability, thermodynamically validating that HA induces a more structurally rigid, energy-demanding barrier against the denaturation of SC.

Considered together, the MD, FTIR, XRD and thermal data show that HA and SC associate through strong electrostatic attraction and multi-site hydrogen bonding. These interactions induce a conformational rearrangement of SC and produce a denser intermolecular network, which provides the structural basis for the interfacial and emulsion behaviors examined in the following sections. It should be emphasized that, because the all-atom MD and the FTIR/XRD/DSC measurements were performed at a single representative complexation condition without explicit pH titration, these atomistic and spectroscopic observations are interpreted here as the mechanistic origin of the network. Specifically, they identify the multi-site hydrogen bonding and the β-sheet–enriched, densified packing that account for the measured rise in interfacial and bulk elasticity, rather than serving as pH-resolved quantitative predictors of the bulk phase behavior. The quantitative coupling across the molecular, interfacial and macroscopic length scales is therefore established separately, from the pH-resolved experimental descriptors (Section 3.4).

### 3.3. Interfacial Adsorption and Viscoelastic Network Formation

The adsorption behavior of biopolymers at the oil-water interface is a key determinant of emulsion formation and stability. As shown in Figure 4f, both SC and HA-SC systems exhibited a rapid drop in interfacial tension during the early adsorption stage, followed by a slower approach to equilibrium. The initial sharp decrease results from the rapid diffusion and adsorption of amphiphilic molecules onto the freshly created interface, while the slower second stage reflects molecular rearrangement and consolidation of the interfacial film [43]. The HA-SC complex consistently produced a lower interfacial tension than SC alone throughout adsorption, and the addition of 0.125% HA reduced the equilibrium tension from 20.87 to 19.78 mN/m. This improved interfacial activity probably arises from HA-SC complex formation, which facilitates the adsorption process and results in a lower equilibrium interfacial tension [14]. Comparable enhancements have been reported for other protein-polysaccharide pairs [42].

**Figure 5:**
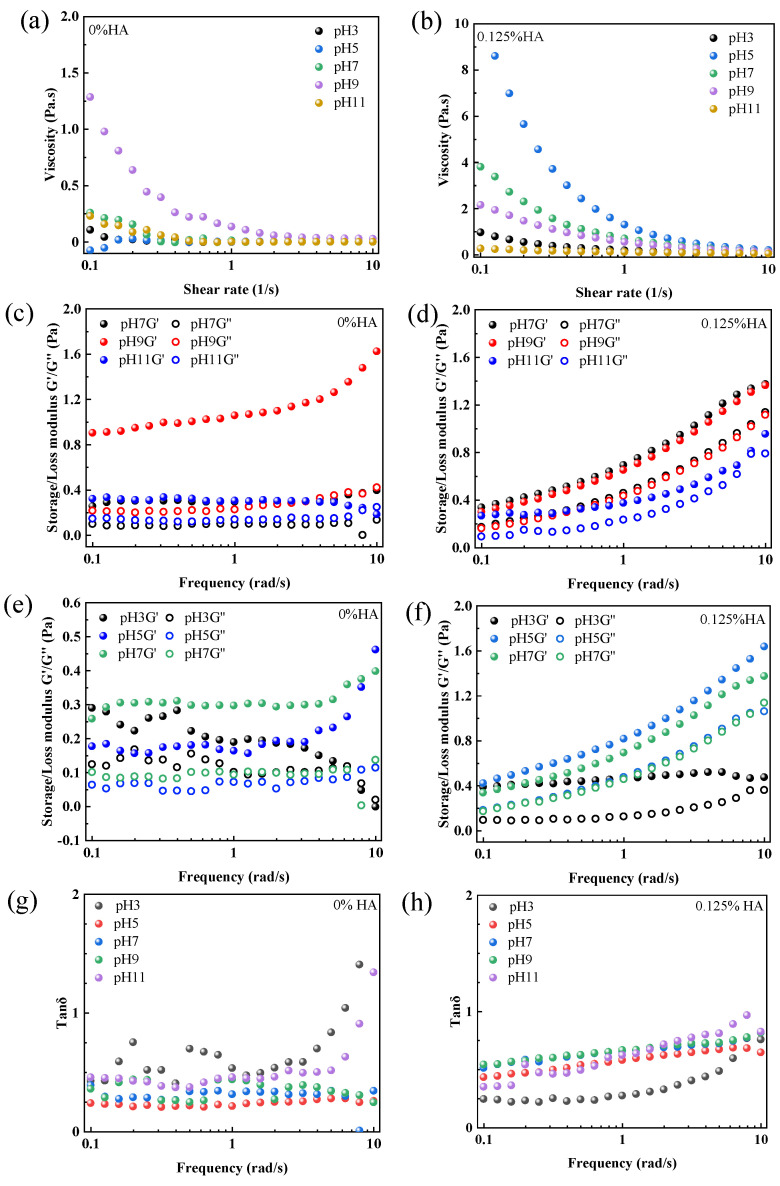
Effect of pH on the bulk rheological behavior of the emulsions at 25 °C: (**a**,**b**) apparent viscosity as a function of shear rate for the (**a**) SC and (**b**) HA–SC emulsions; (**c**–**f**) frequency dependence of the storage modulus (G′) and loss modulus (G″) obtained from small-amplitude oscillatory shear for the SC and HA–SC emulsions, respectively; and (**g**,**h**) tanδ for the SC and HA–SC emulsions.

These enhanced interfacial adsorption characteristics were accompanied by a distinct change in the bulk rheological response of the emulsions. Both SC and HA-SC emulsions showed pronounced shear thinning across most pH conditions (Figure 5a,b), reflecting the disruption and rearrangement of droplet-associated network structures under flow. The shear behavior was strongly pH-dependent. For SC emulsions, the viscosity dropped sharply near pH 5.0 because the system approached the protein pI, where weakened electrostatic repulsion permitted extensive flocculation and partial destabilization [3,37]. The HA-SC emulsions instead reached their highest viscosity at pH 5.0. While macroscopic viscosity alone does not directly prove the underlying driving force, this rheological peak strongly correlates with the electrostatic interactions and structural rearrangements confirmed earlier in our study. Specifically, our ζ-potential measurements (Figure 1a) and FTIR analysis (Figure 4a) demonstrated robust electrostatic attraction and hydrogen bonding between the biopolymers near this pH. Consequently, we propose that the association between oppositely charged HA and SC moieties reinforces the three-dimensional composite network structure under these conditions, leading to the observed maximum in viscosity [41].

Dynamic oscillatory measurements showed that the storage modulus G′ remained higher than the loss modulus G″ over the whole frequency range examined (Figure 5c–f). To quantitatively evaluate the strength and nature of this structured network, the loss tangent (tanδ = G″/G′) was analyzed (Figure 5g,h). For the HA-SC composite emulsions, particularly near pH 5.0, the tanδ values were maintained within the range of 0.44 to 0.69. Values of tanδ < 1, combined with a mild frequency dependence of the moduli, provide quantitative evidence of an elasticity-dominated weak physical gel network, where non-covalent interactions (such as hydrogen bonding and electrostatic attraction) transiently crosslink the droplets. Compared with SC alone, the HA-SC emulsions exhibited substantially higher viscoelastic moduli, indicating that the composite network in the continuous phase increased droplet–droplet interactions and reinforced the overall emulsion structure. The stronger viscoelastic response is in good agreement with the more intermolecular packing inferred from FTIR and XRD analyses.

**Figure 6:**
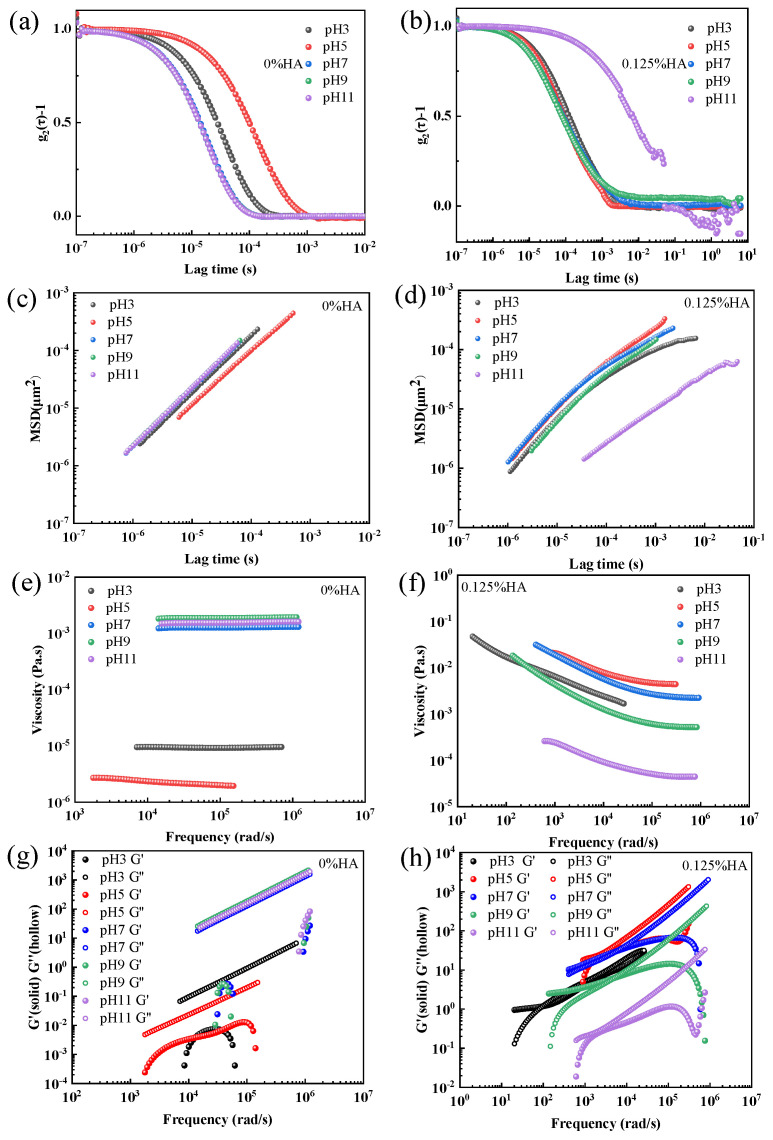
Effect of pH (3.0–11.0) on the diffusing-wave-spectroscopy (DWS) microrheological behavior of the emulsions at 25 °C: (**a**,**b**) intensity autocorrelation function, g_2_(τ) − 1, versus lag time; (**c**,**d**) mean-square displacement (MSD) of the droplets versus lag time; (**e**,**f**) microviscosity as a function of frequency; and (**g**,**h**) the elastic and viscous indices as a function of frequency. Panels (**a**,**c**,**e**,**g**) correspond to the SC emulsion and panels (**b**,**d**,**f**,**h**) to the HA–SC emulsion.

Microrheological measurements clarified how HA influences droplet dynamics on a local scale. The intensity autocorrelation functions of HA-SC emulsions decayed much more slowly than those of SC emulsions (Figure 6a,b), consistent with restricted Brownian motion of droplets within the composite network. In line with this finding, all HA-SC systems produced MSD slopes below 1 (Table 3), characteristic of sub-diffusive behavior. The variation in these MSD slopes across the tested pH range provides critical insight into the local microenvironment, specifically reflecting shifts in the droplet interaction potential and the resulting ‘cage dynamics’.

In dense or complex colloidal systems, a dispersed droplet is temporarily trapped within a microstructural ‘cage’ formed by neighboring particles and the surrounding HA-SC viscoelastic matrix. The stiffness and physical constraint of this cage are fundamentally dictated by the inter-droplet interaction potential. Near the isoelectric point (e.g., pH 5.0), the partial neutralization of charges drastically diminishes the electrostatic repulsive energy barrier. This deepens the attractive potential well—driven synergistically by HA-SC multi-site hydrogen bonding and electrostatic complexation—leading to a highly interconnected, elasticity-dominated weak gel. Within this robust structural cage, droplets experience pronounced steric confinement, restricting their ability to escape the local network. This translates to the sub-diffusive MSD slopes observed (e.g., 0.731 at pH 5.0), which reflect the localized elastic relaxation of droplets within a homogeneous matrix.

Interestingly, the MSD slopes varied significantly at extreme pH conditions. For instance, the steepest drop in the MSD slope (0.554 at pH 11.0) suggests an extremely restricted, solid-like local environment. At this highly alkaline pH, the deacylation of HA and the disruption of the composite network alter the interaction potential towards destabilization. This likely triggers localized crowding or structural jamming of the droplets prior to macroscopic phase separation, tightening the cage effect through close-packing rather than via a homogeneous biopolymer network. Therefore, the variations in microrheological parameters effectively capture how pH-responsive molecular interactions scale up to redefine the physical boundaries of the colloidal cage, thereby governing the macroscopic stability of the emulsions. We attribute this restricted mobility primarily to the formation of a dense, interconnected HA-SC network, which significantly increases the local microviscosity of the continuous phase and kinetically traps the droplets [44].

The HA-SC emulsions also produced higher microviscosity values and a stronger frequency dependence than SC emulsions (Figure 6g,h). The elevated G″ values observed near pH 5.0 in particular point to vigorous microscopic network interactions and improved structural integrity. Crucially, the microrheological dynamics correlate strongly with the bulk macroscopic rheology, bridging the structural properties across different length scales. While bulk rheology captures the overall macroscopic elasticity of the emulsion, the DWS-derived microviscosity specifically probes the localized viscoelastic environment experienced by the droplets. The elevated microviscosity and restricted mean square displacement (MSD) slopes observed for the HA-SC emulsions (Table 3) closely mirror the macroscopic enhancement in bulk viscosity and storage modulus (Figure 5). This strong correlation confirms that the HA-SC association does not merely thicken the continuous phase on a macro level, but actively constructs a homogeneous, multiscale interconnected network. Consequently, this network tightly confines the local microenvironment around individual droplets, physically trapping them to suppress Brownian motion and macroscopic phase separation. Together, these findings demonstrate that HA-mediated association reinforces both interfacial adsorption and bulk viscoelasticity, suppressing droplet mobility and supporting overall emulsion stability.

### 3.4. pH-Responsive Stability and Microstructural Evolution of Emulsions

**Figure 7:**
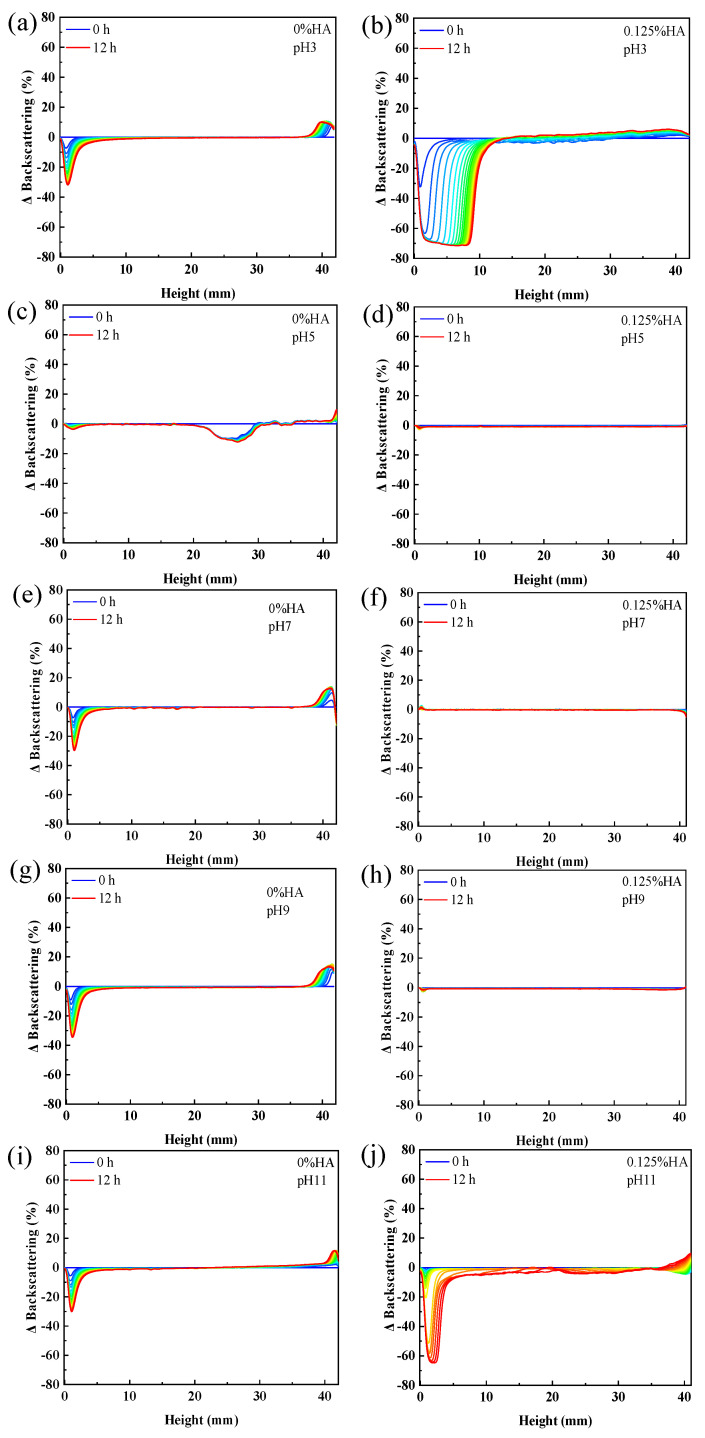
Effect of pH on the multiple-light-scattering profiles of the emulsions recorded by Turbiscan analysis: backscattered-light intensity as a function of sample height for the (**a**,**c**,**e**,**g**,**i**) SC and (**b**,**d**,**f**,**h**,**j**) HA–SC emulsions, with each panel pair corresponding to one of the five pH values examined (3.0, 5.0, 7.0, 9.0, and 11.0).

The macroscopic stability of the emulsions was assessed using Turbiscan analysis, particle size measurements and CLSM observations. As shown in Figure 7, SC emulsions destabilized markedly across the whole pH range, with the upper backscattering signal rising and the bottom signal falling, behavior that signals creaming and the appearance of a clarified bottom phase [37]. The most severe instability occurred at pH 5.0, where a substantial decrease in the middle backscattering intensity revealed extensive droplet aggregation and coalescence, in keeping with the well-documented loss of electrostatic repulsion close to the casein pI [3].

In contrast, the HA-SC emulsions showed only minor backscattering fluctuations between pH 5.0 and 9.0, evidence of much improved physical stability. The almost overlapping initial and final scanning profiles in this pH window indicate that HA significantly limits droplet migration and aggregation under neutral and mildly acidic to alkaline conditions. Stable emulsion systems generally maintain a steady transmitted and backscattered light profile, so the response observed here further confirms that HA appreciably broadens the pH stability window of SC-stabilized emulsions [20]. Outside this window, namely at pH 3.0 and 11.0, noticeable destabilization still developed. At pH 3.0, this destabilization is likely driven by the protonation of HA’s glucuronate residues (pKa ≈ 3.4). Based on previous literature, we hypothesize that this protonation suppresses the electrostatic complexation between HA and the net-positively charged SC, a process that could potentially be accompanied by the partial acid hydrolysis of glycosidic bonds in HA [14]. At pH 11.0, the observed macroscopic instability may be attributed to structural changes in the polysaccharide. Previous studies have demonstrated that highly alkaline conditions can cleave the O-glyceryl and O-acetyl substituents of HA (i.e., deacylation) [45]. While not directly measured in the present study, we postulate that such reactions could alter the conformation of HA and consequently weaken the composite interfacial/continuous network.

**Figure 8:**
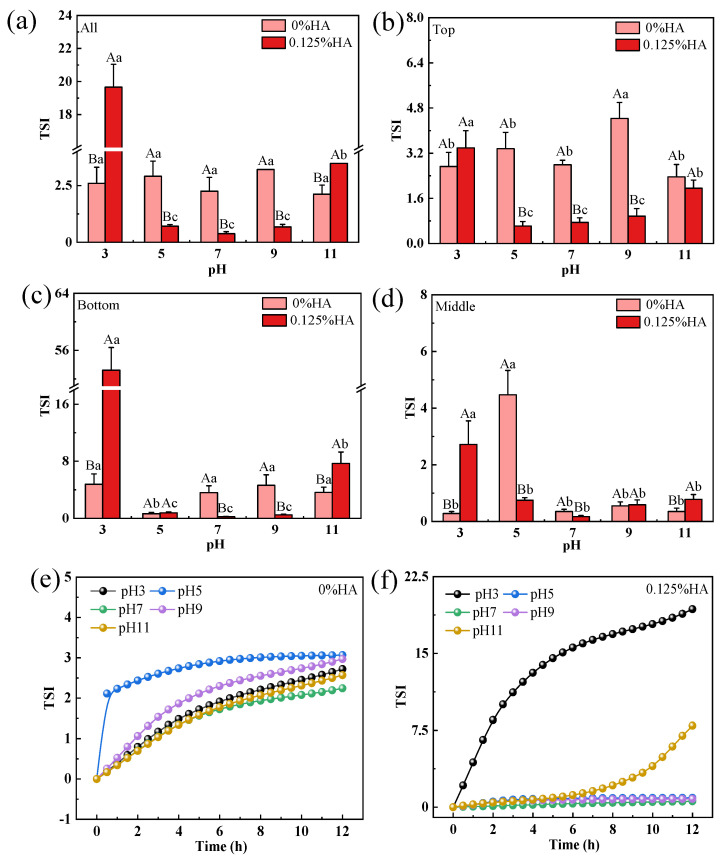
Influence of pH on the Turbiscan Stability Index (TSI) of the SC and HA–SC emulsions: TSI calculated over (**a**) the whole sample (global), (**b**) the top, (**c**) the bottom, and (**d**) the middle zones of the measurement cell; and the time-resolved TSI kinetic curves (12 h, 25 °C) of the (**e**) SC and (**f**) HA–SC emulsions. Bars indicate standard error. Different uppercase letters indicate significant differences within a group, while different lowercase letters indicate differences between groups (*p* < 0.05).

The TSI values quantitatively confirmed the improved stability of the HA-SC emulsions (Figure 8a–f). Compared with SC emulsions, the global TSI fell sharply between pH 5.0 and 9.0, indicating that creaming, clarification and droplet growth were all suppressed. Local TSI analysis showed similar improvements in the top, middle and bottom regions, especially close to the protein pI. The flatter TSI evolution curves of the HA-SC emulsions indicate markedly suppressed incipient destabilization over the measured period; longer-term shelf-life validation is identified as future work. However, while the current study establishes the mechanisms governing the physical stability of these systems, further studies are warranted to evaluate their chemical robustness. Specifically, assessing the oxidative stability of the encapsulated lipids over long-term storage is crucial. Evaluating this oxidative stability alongside the emulsion’s performance within complex food matrices will be a necessary next step to bridge the gap between these fundamental laboratory findings and practical industrial applications.

**Figure 9:**
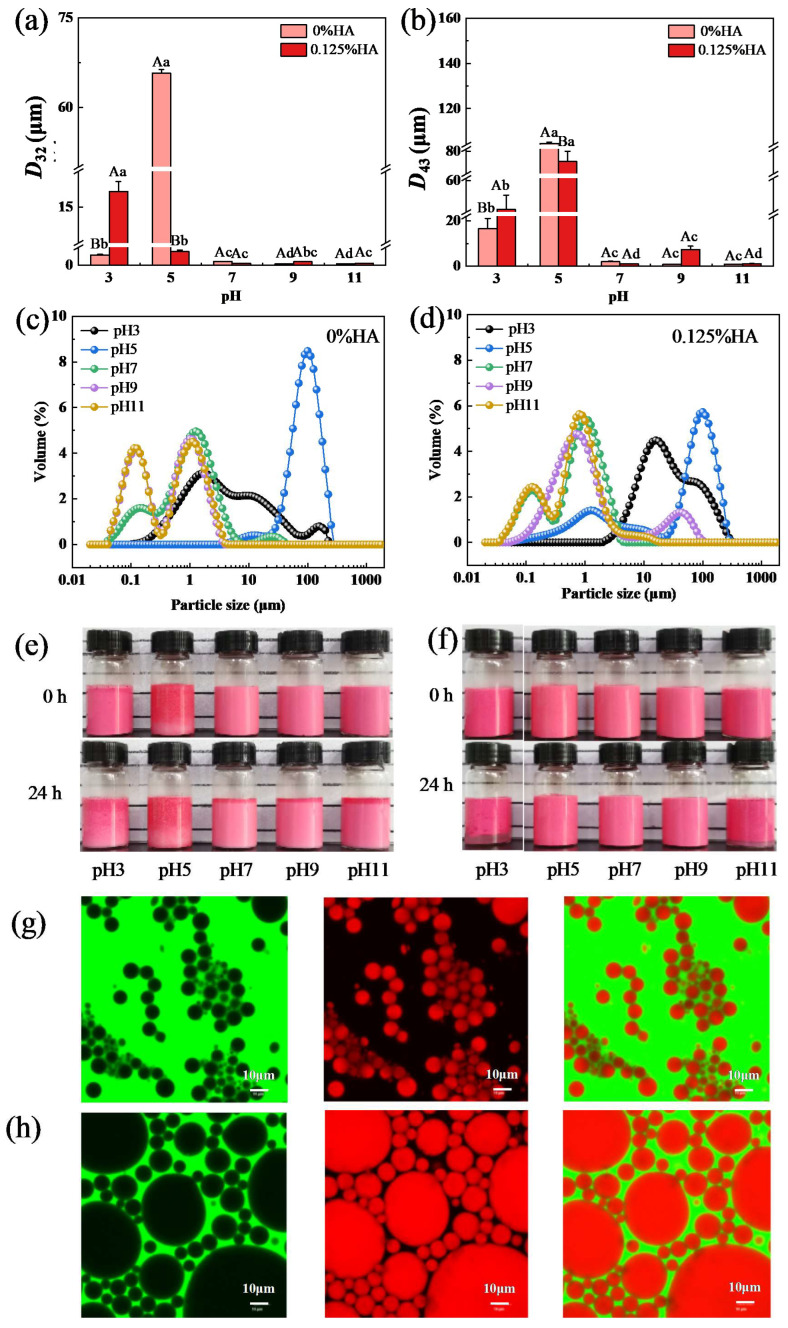
Effect of pH on the droplet characteristics and microstructure of the SC and HA–SC emulsions: (**a**) surface-weighted mean diameter (*D*_32_); (**b**) volume-weighted mean diameter (*D*_43_); (**c**,**d**) droplet-size distributions of the (**c**) SC and (**d**) HA–SC emulsion. (**e**,**f**) Digital photographs of the visual appearance and (**g**,**h**) confocal laser scanning microscopy (CLSM) images of the SC and HA–SC emulsions (oil phase stained with Nile Red, protein phase with FITC; CLSM images acquired at pH 5.0). Bars indicate standard error. Different uppercase letters indicate significant differences within a group, while different lowercase letters indicate differences between groups (*p* < 0.05).

Particle size measurements paralleled the Turbiscan results (Figure 9a,b). Both *D*_32_ and *D*_43_ rose and then fell with pH, peaking near pH 5.0. Under this condition the SC emulsions contained extremely large droplets, the consequence of severe flocculation driven by charge neutralization. HA addition reduced *D*_32_ and *D*_43_ by 96.52% and 27.90%, respectively, demonstrating that the polysaccharide effectively curbs droplet aggregation through combined electrostatic and steric mechanisms. The pure SC emulsion produced a bimodal distribution at pH 5.0 and 11.0 and a trimodal distribution at the other pH values examined (Figure 9c). The HA-SC emulsion, by comparison, retained a bimodal distribution across the entire pH range tested (Figure 9d) Such multimodal patterns are commonly observed in oil-in-water emulsions and usually arise from droplet aggregates formed during homogenization that are difficult to disperse fully by mechanical shear [3].

The improved emulsion stability was also evident from macroscopic appearance and CLSM observations (Figure 9e–h). SC emulsions creamed rapidly under acidic conditions, in particular near pH 5.0, while HA-SC emulsions remained visually homogeneous throughout the early storage stage across a wide pH range. CLSM further showed that SC emulsions contained large, irregularly distributed droplets together with obvious aggregates, while HA-SC emulsions presented smaller and more uniformly dispersed droplets.

The improved microstructural stability of the HA-SC emulsions results from the combined effects of bulk-phase reinforcement and interfacial restructuring. Specifically, the HA-SC complex formed a spatially interconnected network in the continuous phase accompanied by accelerated interfacial adsorption as reflected in the reduced interfacial tension measured here. This accelerated adsorption, together with the interfacial rheology behavior reported for SC/HA films by Farooq et al. [14], worked synergistically with the continuous phase network to limit droplet mobility and inhibit coalescence. Thus, molecular assembly and macroscopic emulsion behavior are coupled; to demonstrate this coupling quantitatively rather than descriptively, the pH-resolved descriptors of each length scale were cross-correlated, as analyzed below.

**Figure 10:**
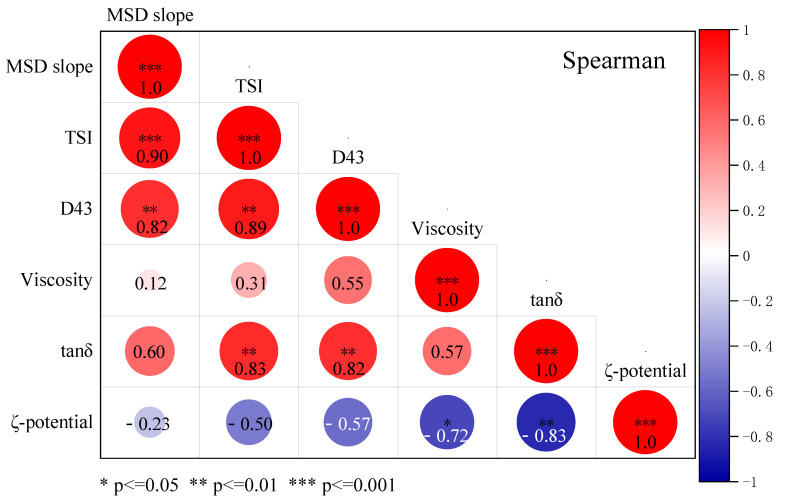
Spearman rank-correlation matrix among the pH-resolved descriptors of the HA–SC emulsion, spanning the molecular (ζ-potential), interfacial/network (network elasticity), macroscopic (Turbiscan Stability Index and droplet diameter) and local droplet-dynamics (DWS mean-square-displacement slope) scales. The size of the circles is proportional to the absolute value of the correlation coefficient. Asterisks denote statistical significance levels (* *p* < 0.05, ** *p* < 0.01, *** *p* < 0.001). The correlation coefficient values inside the circles are displayed in black or white font purely for visual contrast and readability against the background colors.

To demonstrate the coupling across length scales quantitatively rather than descriptively, the pH-resolved descriptors representing the molecular charge state (ζ-potential), the interfacial and continuous-phase network (network elasticity), the macroscopic stability (Turbiscan Stability Index and droplet diameter) and the local droplet dynamics (DWS mean-square-displacement slope) were cross-correlated (Figure 10). Given the limited number of pH levels and the strong leverage of the near-isoelectric extreme on a linear fit, Spearman rank coefficients were adopted for inference. The statistically significant associations formed a connected chain spanning the three length scales: the molecular charge state co-varied monotonically with the interfacial network descriptors (ρ = 0.82–0.90, *p* < 0.01), which in turn tracked the macroscopic stability descriptors (ρ = 0.82–0.83, *p* < 0.01); both were inversely and significantly coupled to droplet mobility (MSD slope; ρ = −0.72 to −0.83, *p* < 0.05–0.01). This provides explicit quantitative evidence that the molecular event of pH-driven charge modulation propagates monotonically through interfacial network reinforcement to droplet-scale confinement and macroscopic stability. Consistent with a mechanistically delimited interpretation, droplet mobility correlated with the proximal network descriptors but not with the distal charge state (ρ = −0.23, n.s.), as expected when droplet dynamics are governed by the local network rather than directly by molecular charge. Because this molecular-to-network step is non-linear around the isoelectric point, it is additionally substantiated mechanistically by the MD and FTIR/XRD evidence (multi-site hydrogen bonding and β-sheet–enriched densification; Section 3.2). Finally, this micro-to-macro mapping is monotonic only within the stable window (pH 5.0–9.0), and the alkaline and acidic extremes were therefore treated as a distinct destabilization regime and excluded from the in-window correlation: at pH 11.0 the lowest MSD slope (0.554) reflects structural jamming and close-packing accompanying HA deacylation rather than a homogeneous protective network, while at pH 3.0 protonation limits HA–SC complexation, so in both cases the confinement signature is decoupled from genuine colloidal stabilization—a distinction the present framework explicitly captures.

In summary, HA effectively compensated for the intrinsic instability of SC near its isoelectric point and broadened the pH tolerance of the resulting emulsions. The findings highlight HA-SC complexes as robust food-grade stabilizers for emulsion systems exposed to varied pH environments and are consistent with the growing recognition of HA as a versatile functional ingredient in dairy and emulsion applications. It is worth noting that while the 10% oil fraction used in this study serves as an idealized mechanistic model, allowing for the clear elucidation of HA-SC interfacial assembly without the confounding rheological effects of droplet crowding, it is directly applicable to specific realistic food systems. An oil fraction of 10% is highly representative of liquid and semi-liquid emulsion products, such as fortified functional beverages, plant-based milk alternatives, and dairy-based drinks. The robust pH tolerance demonstrated by the HA-SC complexes in this model system establishes a fundamental physical-stability foundation for formulating acid-tolerant liquid products. Because gastrointestinal fate is essential for functional applications, and building upon the recognized digestive behaviors of gellan gum and caseinate systems [8,15], future investigations must explicitly target the in vitro gastrointestinal stability and bioaccessibility of these specific multiscale networks. Furthermore, it will be necessary to extrapolate these stabilization mechanisms to high-internal-phase emulsions (HIPEs) or complex food matrices like mayonnaise and semi-solid dressings, where volume-exclusion effects and bulk rheology play more dominant roles.

**Figure 11:**
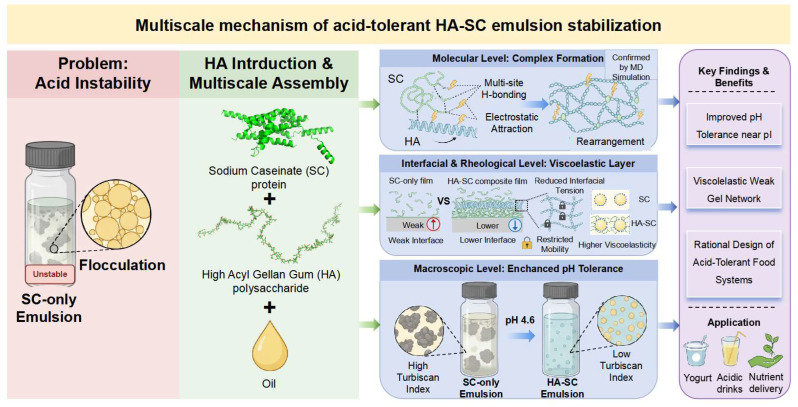
Schematic illustration of the pH-responsive multiscale stabilization mechanisms of high acyl gellan gum (HA)–sodium caseinate (SC) emulsions. Arrows denote the logical flow of the stabilization process. Upward red and downward blue arrows indicate an increase and decrease in interfacial properties, respectively.

To systematically conceptualize the findings discussed above, a schematic model of the pH-responsive multiscale stabilization mechanism for the HA-SC emulsions is proposed in Figure 11. At pH values near the isoelectric point of SC, the strong electrostatic attraction and multi-site hydrogen bonding between the oppositely charged HA and SC induce a localized protein conformational rearrangement. This robust non-covalent complexation not only accelerates biopolymer interfacial adsorption but also constructs a dense, elasticity-dominated weak gel network in the continuous phase. Consequently, this multiscale structural reinforcement traps the dispersed oil droplets within a robust viscoelastic “cage”, effectively restricting droplet mobility and suppressing collision-induced coalescence. Through this pathway, the multiscale interfacial engineering broadens the operational pH window of SC-stabilized systems, granting the emulsions exceptional macroscopic stability against severe acidic stress.

## 4. Conclusions

This study mapped the pH dependent assembly of high acyl gellan gum (HA) and sodium caseinate (SC) and linked it to the multiscale stability of oil in water emulsions. Incorporating HA reshaped the surface charge distribution of SC, shifting the apparent isoelectric point of the complex from pH 4.6 to approximately 4.2. Driven by electrostatic attraction and multi-site hydrogen bonding, SC underwent conformational rearrangement, with β sheet content rising from 32.02% to 42.75%, which promoted a dense and intermolecular network. At the oil water interface, complexation accelerated biopolymer adsorption and lowered the equilibrium interfacial tension to 19.78 mN/m. Meanwhile, the HA–SC composite formed an elasticity dominated weak gel that physically restricted droplet mobility, as indicated by a sub diffusive mean square displacement slope of 0.731 at pH 5.0. Cross scale rank correlation analysis confirmed that the molecular charge state varied monotonically with the interfacial and macroscopic stability descriptors (ρ = 0.82–0.90, *p* < 0.01) and was inversely coupled to droplet mobility (ρ = −0.72 to −0.83). Consequently, HA compensated for the intrinsic instability of SC near its isoelectric point, reducing the surface weighted mean diameter (*D*_32_) by 96.52% relative to pure SC emulsions at pH 5.0 and suppressing macroscopic phase separation across a broad pH continuum (pH 5.0–9.0).

These findings were obtained in a defined model system (fixed 0.2% *w*/*v* SC and 0.125% *w*/*v* HA, 10% *v*/*v* oil) with simplified, purely aqueous molecular dynamics simulations that serve as a qualitative illustration of localized noncovalent docking rather than a quantitative predictor of bulk behavior. Future work should therefore explore broader SC/HA mass ratios and higher oil fractions such as high internal phase emulsions (HIPEs), evaluate in vitro gastrointestinal stability and bioaccessibility, and validate performance within complex food matrices (e.g., mayonnaise, semi solid dressings, fermented beverages) under real world processing stresses.

## Figures and Tables

**Table 1:** Parameters of the simulated system.

System	Casein Molecule	High Gellan Gum Molecule	Temperature (K)	Simulation Time
Casein-high acyl gellan gum	1	1	298.15	100 ns

**Table 2:** The relative content of secondary structure in SC and HA-SC complex coacervates. Means annotated with different letters within the same column are significantly different (*p* < 0.05).

Samples	Wavenumber (cm^−1^)	Assignment	Relative Contents (%)
SC	1615–1637, 1682–1700	β-sheet	32.02 ± 2.86 ^b^
	1637–1645	random coil	20.09 ± 1.90 ^a^
	1646–1664	α-helix	31.75 ± 2.94 ^a^
	1664–1681	β-turn	16.14 ± 1.50 ^b^
HA-SC	1615–1637, 1682–1700	β-sheet	42.75 ± 4.03 ^a^
	1637–1645	random coil	13.18 ± 1.95 ^b^
	1646–1664	α-helix	22.12 ± 0.91 ^b^
	1664–1681	β-turn	21.95 ± 0.28 ^a^

**Table 3:** The effect of pH on the MSD slope of SC single emulsion and HA-SC composite emulsion.

Sample	pH 3	pH 5	pH 7	pH 9	pH 11
SC	1.002	0.926	1.008	1.007	1.012
HA-SC	0.599	0.731	0.663	0.739	0.554

## Data Availability

The original contributions presented in this study are included in the article. Further inquiries can be directed to the corresponding author.
